# Beyond Individual Tests: Youths’ Cognitive Abilities, Basic Reading, and Writing

**DOI:** 10.3390/jintelligence12110120

**Published:** 2024-11-20

**Authors:** Jacqueline M. Caemmerer, Audrey M. Scudder, Timothy Z. Keith, Matthew R. Reynolds

**Affiliations:** 1 Department of Educational Psychology, University of Connecticut, Storrs, CT 06268, USA; audrey.scudder@uconn.edu; 2 Department of Educational Psychology, University of Texas at Austin, Austin, TX 78712, USA; tim@keithresearch.com; 3 Department of Educational Psychology, University of Kansas, Lawrence, KS 66045, USA; mreynolds@ku.edu

**Keywords:** intelligence, academic, achievement, spelling, moderation, mediation

## Abstract

Broadly, individuals’ cognitive abilities influence their academic skills, but the significance and strength of specific cognitive abilities varies across academic domains and may vary across age. Simultaneous analyses of data from many tests and cross-battery analyses can address inconsistent findings from prior studies by creating comprehensively defined constructs, which allow for greater generalizability of findings. The purpose of this study was to examine the cross-battery direct effects and developmental differences in youths’ cognitive abilities on their basic reading abilities, as well as the relations between their reading and writing achievement. Our sample included 3927 youth aged 6 to 18. Six intelligence tests (66 subtests) and three achievement tests (10 subtests) were analyzed. Youths’ general intelligence (*g*, large direct and indirect effects), verbal comprehension–knowledge (large direct effect), working memory (large direct effect), and learning efficiency (moderate direct effect) explained their basic reading skills. The influence of *g* and fluid reasoning were difficult to separate statistically. Most of the cognitive–basic reading relations were stable across age, except the influence of verbal comprehension–knowledge (Gc), which appeared to slightly increase with age. Youths’ basic reading had large influences on their written expression and spelling skills, and their spelling skills had a large influence on their written expression skills. The directionality of the effects most strongly supported the direct effects from the youths’ basic reading to their spelling skills, and not vice versa.

## 1. Introduction

Intelligence and academic tests are often administered to better understand individuals’ academic performance. Youths’ general intelligence explains more than half of the variation in their standardized achievement test scores and more than a quarter of the variation in school grades (Deary et al. 2007; Kaufman et al. 2012; Roth et al. 2015). Other more specific cognitive abilities also predict youths’ academic performance, to a lesser extent, but there are differential effects across academic domains and the strength of these cognitive–achievement relations may depend on the individual’s age.

The hierarchical Cattell–Horn–Carroll (CHC) taxonomy of cognitive abilities can be used to interpret cognitive–achievement relations. At the top of the CHC model is general intelligence (*g*), which measures general reasoning, problem solving, and learning (Colom et al. 2010). At the second level of the CHC model there are several broad cognitive abilities. General intelligence and broad cognitive abilities operate together in a system of interrelated cognitive abilities (Schneider and McGrew 2018). The broad abilities examined in this study include fluid reasoning (also referred to as fluid intelligence, Gf, the ability to solve novel problems that cannot be answered automatically), verbal comprehension–knowledge (also referred to as crystallized intelligence, Gc, the breadth and depth of acquired cultural knowledge, language, and the knowledge learned inside and outside of school), working memory (also referred to as short-term working memory, Gwm, the ability to mentally hold and manipulate information in immediate awareness), learning efficiency (also referred to as long-term retrieval or long-term memory, Gl, the ability to learn, store, and retrieve new information in long-term memory), visual processing (also referred to as visual spatial abilities, Gv, the ability to solve problems using mental rotations of objects or pattern recognition), and processing speed (Gs, the ability to perform simple, repetitive tasks quickly and accurately). At the lowest levels are a large number of narrow abilities, which subsume test-specific abilities (Schneider and McGrew 2018).

Although some CHC cognitive–achievement relations consistently emerge across studies, there are also inconsistent findings, which have the potential to confuse the interpretation and application of results. One potential explanation for these inconsistencies may be methodological. Typically, studies use a single intelligence test to predict a single achievement test. The use of single tests in isolation means findings are narrowly focused and may not generalize to other tests, particularly because most intelligence tests do not measure the six CHC broad abilities described above, and broad abilities and academic skills are measured by a limited number of subtests per test (Caemmerer et al. 2020; Zaboski et al. 2018). The simultaneous, or cross-battery, analysis of multiple intelligence and multiple achievement tests can include many more subtests per construct and can improve the construct representation in cognitive–achievement studies. To date, there are only two cross-battery cognitive–achievement studies–one study focused on broad writing and math (Caemmerer et al. 2023) and one study focused on broad reading (Flanagan 2000). The purpose of our study is to provide cross-battery evidence for the influence of youths’ cognitive abilities on their standardized basic reading performance, to examine the developmental patterns of cognitive–basic reading relations, and to explore the interrelations between basic reading and writing skills.

### 1.1. Cognitive Influences on Basic Reading Skills

Basic reading, also referred to as word reading skills or code skills (Peng et al. 2019), refers to individuals’ decoding and word recognition skills—sounding out unfamiliar words and the automatic identification of sight words. Reading is a complex skill that involves several cognitive abilities. Three cognitive abilities have shown a consistent influence on youths’ basic reading performance, and these effects transcend the intelligence and academic tests used in these studies. First, children’s general intelligence (*g*) has a large direct influence on their basic reading skills, and an indirect influence when CHC broad cognitive abilities are included (Caemmerer et al. 2018; Canivez et al. 2017; McGill 2015; Woods et al. 2021). Second, children’s verbal comprehension–knowledge (Gc) has a large effect on their basic reading skills (Caemmerer et al. 2018; Elliott et al. 2010; Floyd et al. 2008; Hajovsky et al. 2014; McGrew and Knopik 1993; Niileksela et al. 2016; Oh et al. 2004). The strength of the Gc–basic reading relation may vary by age. Some studies suggest Gc has a stronger influence on adolescents’ basic reading in comparison to younger children (Benson 2008; Hajovsky et al. 2014; McGrew and Knopik 1993; Zaboski et al. 2018), but one study with the Wechsler tests found the Gc–basic reading relation was stable throughout children’s development (Caemmerer et al. 2018). Third, youths’ working memory abilities (Gwm) consistently influence their basic reading skills (Caemmerer et al. 2018; Cormier et al. 2016; Elliott et al. 2010; Floyd et al. 2008; Hajovsky et al. 2014; McGrew and Knopik 1993; Peng et al. 2019). One Woodcock-Johnson Fourth Edition study, however, found a non-significant Gwm–basic reading effect (Niileksela et al. 2016). Whether or not the strength of the Gwm–basic reading relation varies across age is less clear. Some studies suggest Gwm has a stable influence on basic reading skills across children’s development (Caemmerer et al. 2018; Floyd et al. 2008; Hajovsky et al. 2014), but a correlational meta-analysis found the influence was stronger prior to fourth grade (Peng et al. 2019). Children’s general intelligence (*g*), vocabulary and oral expression (Gc), and ability to hold information in their mind while manipulating it (Gwm) all have important influences on their decoding and word recognition skills, but whether these effects are moderated by children’s age is unclear.

The influence of children’s learning efficiency and processing speed are less consistent, and less is known about their potential developmental differences. Children’s learning efficiency (Gl) appears to influence their basic reading skills (Floyd et al. 2008; Hajovsky et al. 2014; McGrew and Knopik 1993), possibly more strongly for younger children, but the Gl–basic reading relation has not been tested frequently. There are conflicting findings related to processing speed which may be attributed to measurement differences. Across the editions of the Woodcock-Johnson tests, children’s processing speed appears to significantly influence their basic reading skills (Floyd et al. 2008; McGrew and Knopik 1993; Niileksela et al. 2016). On the Wechsler Intelligence Scale for Children, Fifth Edition (WISC-V), and the Differential Abilities Scale for Children, Second Edition (DAS-II), processing speed did not influence the children’s basic reading skills, but the DAS-II study found a significant effect for children with low reading skills (Caemmerer et al. 2018; Elliott et al. 2010).

Generally, children’s fluid reasoning (Gf) and visual processing (Gv) abilities do not appear to influence their basic reading skills (Caemmerer et al. 2018; Floyd et al. 2008; Hajovsky et al. 2014; Niileksela et al. 2016; Zaboski et al. 2018), with one Gf exception with the DAS-II (Elliott et al. 2010). One hypothesis is that novel problem-solving abilities (Gf) may exert larger influences on more complex academic skills, such as reading comprehension, rather than basic academic skills (Peng et al. 2019). Additionally, while there does not appear to be a significant Gv–basic relation among typical readers, Gv may be important for children with dyslexia and other reading disabilities (Bellocchi et al. 2017; Elliott et al. 2010).

Although some cognitive abilities have clear consistent cognitive influences on children’s basic reading, several cognitive–basic reading relations are ambiguous due to inconsistent findings. One potential explanation for the inconsistencies is measurement differences; the tests measure cognitive and academic constructs differently. Across the tests, cognitive and academic subtests involve different task demands, stimuli, and response formats. Thus, divergent cognitive–achievement findings may be a result of the unique aspects of the subtests. More generalizable findings may be possible when several cognitive and academic tests are analyzed simultaneously. These joint analyses of multiple tests are referred to as cross-battery analyses. There is one prior cross-battery broad reading study. In the prior study, two intelligence tests, the Wechsler Intelligence Scale for Children, Revised (WISC-R) and Woodcock–Johnson-Revised (WJ-R) Cognitive, and one academic test, the WJ-R Achievement, were simultaneously analyzed. The sample was small and homogeneous and a broad reading variable that combined the children’s basic reading and reading comprehension skills was examined (Flanagan 2000). The cross-battery cognitive–broad reading findings shared some similarities with studies based on single cognitive and academic tests. The children’s verbal comprehension–knowledge had the strongest influence on broad reading. Processing speed also significantly influenced the children’s broad reading skills (Flanagan 2000), which has been found in single test studies with the Woodcock–Johnson test.

### 1.2. Academic Interrelations: Basic Reading and Writing Skills

In addition to the important influences of children’s cognitive abilities on their academic skills, children’s skills in one academic domain can influence their skills in another academic domain. More specifically, children’s reading and writing skills are both language-based skills that are interrelated (Shanahan 2006). Basic reading skills influence basic writing skills, often measured by spelling tasks. More specifically, children’s decoding predicts their spelling, and improvements in decoding and word reading are associated with improvements in spelling (Ahmed et al. 2014; Andersen et al. 2018; Georgiou et al. 2020; Kim and Park 2018). This basic reading–spelling relation occurs in a variety of languages and orthographies (Georgiou et al. 2020). Evidence also supports the reverse direction of effects; children’s spelling predicts their basic reading. Spelling, particularly invented spelling (a child’s early attempts to represent spoken language in written form; Goodrich et al. 2016), uniquely predicted children’s later basic reading skills even after controlling for earlier reading skills (Andersen et al. 2018; Clemens et al. 2014; Hand et al. 2022; McNeill et al. 2023). Furthermore, experimental evidence suggests that spelling interventions result in improvements in reading ability (Martins et al. 2013). A small number of longitudinal studies have tested both directions of effects. Two such studies found that children’s earlier word reading predicted their later spelling (Kim and Park 2018; Schaars et al. 2017), but one study found that young children’s earlier phonological spelling predicted their later word reading (Caravolas et al. 2001). Clearly, basic reading and spelling are related (Berninger and Chanquoy 2012), but more research is needed to clarify the direction of effects between basic reading and spelling.

Children’s basic reading and basic writing skills also appear to influence their advanced writing skills (also referred to as written expression), often measured by sentence and essay writing. Elementary school children’s decoding and word recognition skills at the beginning of the school year predicted their sentence and essay writing skills at the end of the school year, even after controlling for initial writing skills (Andersen et al. 2018; Mäki et al. 2001). Within the not-so-simple view of writing theory, children’s spelling (one aspect of transcription skills, the other being handwriting) influences their sentence and essay writing (text generation) alongside language and self-regulation skills (Berninger and Chanquoy 2012). Across different ages, youth’s spelling skills appear to have a moderate to large influence on their sentence writing and writing quality (Kim et al. 2015; Parkin et al. 2020).

### 1.3. Purpose of This Study

Studies of the effects of children’s cognitive abilities on their academic skills are narrowly focused on single tests analyzed in isolation. Single test studies may contain a limited number of CHC broad cognitive ability factors and a limited number of subtest indicators per CHC broad ability factor (often two or three subtests) and achievement outcome (often one or two subtests). These limitations likely contribute to the inconsistencies observed across studies in some cognitive–achievement relations across tests. Thus, relations are less generalizable to students’ cognitive and achievement abilities more broadly. Another limitation of prior cognitive–achievement studies is many cognitive–achievement findings are based solely on the Woodcock–Johnson Tests of Cognitive Abilities and Achievement (WJ), but several other intelligence tests are frequently used in practice (Benson et al. 2019; Rabin et al. 2016).

Cross-battery studies of cognitive–achievement relations can address these methodological and measurement limitations. A broader understanding of cognitive–achievement relations is possible through the joint analysis of several intelligence and achievement tests, which analyze a large variety of subtests simultaneously. Furthermore, relatively few studies have examined the developmental patterns of cognitive–achievement relations, which affect the generalizability and the interpretation of cognitive–achievement relations. Lastly, examinations of the interrelations of academic skills can test the potential mediators of cognitive—achievement relations and can potentially inform intervention plans.

This study is part of a three-study cross-battery analyses series. Each study used the same samples and tests, but the outcome variables and research questions differed. The purpose of the first study was to test the application of CHC theory to six intelligence tests and establish the cross-battery CHC cognitive model used in this study (Caemmerer et al. 2020). The purpose of the second study was to investigate cross-battery cognitive influences on youth’s broad math and writing skills (Caemmerer et al. 2023).

The current study used the largest cross-battery CHC intelligence model to date (which we described in detail in Caemmerer et al. 2020) to address three research questions:

1. Which cognitive–basic reading relations generalize across six intelligence and three achievement tests?
2. Which cognitive–basic reading relations are moderated by youths’ age?
3. What is the magnitude of effects between youths’ basic reading, spelling, and written expression skills?
  - What is the directionality of effects between youths’ basic reading and spelling skills?

## 2. Materials and Methods

The sample consisted of 3927 youth between the ages of 6 and 18 (*M* = 11.45, *SD* = 3.40) with similar numbers of girls and boys (n = 1959 and 1968, respectively). A total of 60% of the youth were white, 19% were Hispanic, 15% were Black, 5% were missing race and ethnicity data, 4% were identified as other, and 2% were Asian. A total of 31% of the youths’ parents had completed some college or technical program, 29% were high school graduates or earned a General Equivalency Diploma (GED), 25% earned a Bachelor’s degree or higher, 15% completed grade 11 or lower, and 0.3% had missing data for the highest education level of their parents. Pearson Assessments coded the demographic data.

The total sample consisted of seven standardization and linking samples collected by Pearson Assessments (see Table 1). This is the same sample used in the large cross-battery intelligence model and the cross-battery cognitive–broad math and broad writing studies described above (see Caemmerer et al. 2020 and Caemmerer et al. 2023). Most of the youth in the total sample completed two tests (99.60%), but 16 youth completed two intelligence and one achievement test because they participated in more than one standardization or linking sample. Deidentified data were shared with the first author via Pearson Assessments, and the University of Connecticut IRB determined that the use of the data did not meet the criteria for human subjects research.

### 2.1. Measures

Six intelligence tests (66 subtests) and three achievement tests (10 subtests) were analyzed in the cross-battery models. Table 2 lists the subtests and describes the cognitive abilities and academic skills they measured. Age-referenced standardized subtest scores were analyzed.

#### 2.1.1. Cognitive Tests

The Kaufman Assessment Battery for Children, Second Edition (KABC-II) measures five CHC broad abilities: Gf, Gc, Gv, Gwm, and Gl. Sixteen KABC-II subtests were analyzed (*M* = 10, *SD* = 3), completed by participants ages 6 to 18 from three samples (see Table 1 and Table 2). The internal consistency estimates were adequate in the norming sample (α = 0.74–0.93, Kaufman and Kaufman 2004). In the KABC-II XBA sample, the KABC-II and the other cognitive test (WJ III, WISC-III, and WISC-IV) were administered in counterbalanced order; the testing interval between the tests was not specified (Kaufman and Kaufman 2004).

The Wechsler Intelligence Scale for Children (WISC) measures five CHC broad abilities: Gf, Gc, Gv, Gwm, and Gs. Eighteen WISC subtests were analyzed (*M* = 10, *SD* = 3), completed by participants ages 6 to 16 from five samples (see Table 1 and Table 2). The internal consistency estimates were adequate in the norming samples (WISC-V α = 0.80–0.96, Wechsler 2014; WISC-IV α = 0.81–0.91, Wechsler 2003; WISC-III α = 0.69–0.87, Wechsler 1991). In the WISC-V/KABC-II sample, the tests were administered in counterbalanced order with a testing interval of 14–70 days (mean = 22 days; Wechsler 2014).

The Differential Abilities Scale, Second Edition (DAS-II) measures six CHC broad abilities: Gf, Gc, Gv, Gwm, Gl, and Gs. Fourteen DAS-II subtests were analyzed (*M* = 50, *SD* = 10), completed by participants ages 6 to 17 from two samples (see Table 1 and Table 2). The internal consistency estimates were adequate in the norming sample (α = 0.68–0.97, (Elliott 2007)). In the DAS-II/WISC-IV sample, all the participants completed the DAS-II first with a testing interval of 1–72 days (mean = 20.5 days; (Elliott 2007)).

The Woodcock–Johnson Tests of Cognitive Abilities, Third Edition (WJ III) measures the most CHC broad abilities, and six CHC broad abilities were included in our study: Gf, Gc, Gv, Gwm, Gl, and Gs. Eleven WJ III subtests were analyzed (*M* = 100, *SD* = 15), completed by participants ages 7 to 16 from one sample (see Table 1 and Table 2). The internal consistency estimates were adequate in the norming sample (α = 0.74–0.94, (Woodcock et al. 2001)).

#### 2.1.2. Academic Achievement Tests

Four Kaufman Test of Educational Achievement, Second Edition (KTEA-II) subtests were analyzed (*M* = 100, *SD* = 15), completed by participants ages 6 to 18 from one sample (see Table 1 and Table 2). The split-half reliability estimates were adequate in the norming sample (α = 0.89–0.97, Kaufman and Kaufman 2004). The KABC-II and KTEA-II co-normed sample was used. The two tests were given to the participants in counterbalanced order and the testing interval was not specified (Kaufman and Kaufman 2004).

Six Wechsler Individual Achievement Test (WIAT) subtests were analyzed (*M* = 100, *SD* = 15), completed by participants ages 6 to 17 from three samples (see Table 1 and Table 2). The reliability estimates were generally above 0.90 in both of the WIAT norming samples (Breaux 2010; Wechsler 2005). In the WIAT-II/DAS-II sample, all the participants completed the DAS-II first and the testing interval was 0–56 days (mean = 4.9–10.1 days; (Elliott 2007)). In the WIAT-II/WISC-IV sample, most of the participants completed the WISC-IV first and the testing interval was 0–39 days (mean = 12 days). The administration order for the WIAT-III/WISC-V sample was not specified, but the testing interval was 0–59 days (mean = 16 days; Wechsler 2014).

### 2.2. Linking the Samples

Pearson Assessments collected the seven samples for the purposes of test standardization (KABC-II/KTEA-II), convergent validity between two or more intelligence tests (KABC-II XBA, WISC-IV/DAS-II, WISC-V/KABC-II), and predictive validity between an intelligence and achievement test (DAS-II/WIAT-II, WISC-IV/WIAT-II, WISC-V/WIAT-III; see Table 1). In one sample, the KABC-II XBA, all 350 participants completed the KABC-II and a second intelligence test (WJ III, WISC-III, and WISC-IV). The KABC-II XBA data resembled a planned missingness design because the participants did not complete all the tests in the study, but they all completed one test referred to as the linking test (i.e., KABC-II). Planned missingness designs allowed the researchers to increase the number of variables or breadth of the data in a study (McArdle 1994). Missingness was intentionally planned for and the incomplete data were distributed across the participants to minimize the time and financial demands and fatigue related to administering several tests (McArdle 1994; Enders 2022). The other six samples in our study did not share the same linking test and missingness was not intentionally planned. Rather, our study samples were “linked” to each other through the tests they shared in common (see Table 1), which resulted in a wide breadth of tests and a large number of indicators per each latent construct. Refer to Caemmerer and colleagues (2020) for further details.

### 2.3. Data Analyses

#### 2.3.1. Missing Data

Data from the same intelligence and achievement tests were combined across the seven datasets. Every participant in our combined dataset had missing data because no one completed six intelligence and three achievement tests. To evaluate how missingness relates to the variables in our study, three missing data mechanisms must be considered (Enders 2022; Rubin 1987). Missing completely at random (MCAR) means there is no association between the test scores and the cause of the missingness. Missing at random (MAR) means the missing data are also not related to test scores, but that the missingness is related to other measured variables in the model; data are considered MAR when there is no relation between the missing data and the incomplete outcome variable (Enders 2022). The third mechanism, missing not at random, means missingness is related to the test scores on the outcome variable (Enders 2022). In our study, data were missing due to our methodological approach that combined the subtest scores across seven samples. Most of the datasets were collected with the intention of providing data on two tests for each participant. Thus, the cause of missingness was not due to the youths’ scores on the outcome variables. For this reason, missingness was likely MCAR or MAR because the cause of the missingness was unrelated to children’s scores on the intelligence and achievement tests.

Missing data were handled with maximum likelihood (ML) estimation. ML is an iterative process that maximizes the data available by predicting incomplete variables from observed data and accounting for missing data patterns in the standard errors (Enders 2022). ML uses incomplete data to improve the accuracy and power of the estimation process (Enders 2022; Schafer and Graham 2002). When used with MCAR or MAR data, ML produces unbiased parameter estimates in correctly specified models (McArdle 1994; Rubin 1987).

Within our combined dataset, there were 83 missing data patterns in the basic reading and basic reading plus spelling models, 87 missing data patterns in the spelling plus written expression model, and 92 missing data patterns in the basic reading plus written expression model. Approximately 68% of the total sample completed the KABC-II and KTEA-II, 30% completed an edition of the WISC, 28% completed an edition of the WIAT, 14% completed the DAS-II, and 2% completed the WJ III.

#### 2.3.2. Analysis Step 1: Invariance Tests

As precursors to the cross-battery cognitive–achievement and age moderation models, several tests of measurement invariance were previously completed via SPSS Amos Version 23.0 (Arbuckle 2015; see Caemmerer et al. 2020, 2023 for further details). Measurement invariance was supported for tests with multiple editions (e.g., WIAT-II and -III, WISC-III, -IV, -V) and for tests that were completed by multiple samples of participants (e.g., KABC-II, WISC-IV, WISC-V, DAS-II, WIAT-II, see Table 1). Data from the same tests were merged across the samples, and the data from the same subtests were merged across multiple editions of the same test and referred to broadly by the test name (e.g., WIAT for the three WIAT-II and WIAT-III merged subtests; Caemmerer et al. 2020, 2023). Additionally, previous studies established invariance across youths’ age for the nine tests (see Caemmerer et al. 2023 for further details).

#### 2.3.3. Analysis Step 2: Cross-Battery Cognitive–Basic Reading Models

Mplus version 8.11 (Muthén and Muthén 2017) and the maximum likelihood estimator were used to analyze the cross-battery cognitive–achievement structural equation models. The higher order CHC model included *g* and six broad ability latent factors. Verbal–comprehension knowledge, fluid reasoning, and visual processing were estimated by 15 subtests, working memory was estimated by 12 subtests, and learning efficiency and processing speed were estimated by 8 subtests (see Caemmerer et al. 2020 for information about how the CHC intelligence model was established). The latent basic reading factor was estimated by four subtests.

Fluid reasoning’s residual variance was constrained to zero in all of the models in our study. The same constraint was made in the previously established cross-battery intelligence model (Caemmerer et al. 2020) and broad reading and writing study (Caemmerer et al. 2023), which found that fluid reasoning’s (Gf) unique variance was not statistically significant from zero and Gf’s standardized factor loading on *g* was very high (β = 0.99). Other studies have also found that fluid reasoning and *g* are statistically indistinguishable (Carroll 2003; Gustafsson 1984; Hajovsky et al. 2018; Reynolds et al. 2013). Because this constraint collapsed fluid reasoning and *g* on each other, the paths from fluid reasoning can be interpreted as effects of *g*, or vice versa. Supplemental analyses demonstrated the results were identical if a direct path from *g* replaced the Gf direct path.

As a first step, the direct paths from the six CHC broad abilities and a total indirect effect from *g*, mediated by the broad abilities, to basic reading were tested. Second, all the non-statistically significant paths (*p* > .05) were simultaneously deleted from the model (Caemmerer et al. 2023; Niileksela et al. 2016). In a separate model, the direct influence of *g* was tested; thus, only one direct path was examined.

#### 2.3.4. Analysis Step 3: Age Moderation of Cognitive–Basic Reading Relations

Moderation tests examined if the influence of children’s broad cognitive abilities on their basic reading performance depended on their age. Potential interactions between the five broad abilities and age, and both continuous variables, were tested individually in the separate models. Interactions between fluid reasoning and age were not tested because fluid reasoning’s residual variance was constrained to zero. Cross products between age and the broad cognitive abilities were created via the “XWITH” procedure in Mplus. Each interaction model included the (1) cross product (e.g., age multiplied by learning efficiency), (2) the direct effects from age and the broad cognitive ability in the cross product regardless of the statistical significance of these paths, and (3) the other broad cognitive abilities that statistically significantly predicted basic reading in step two of the analysis. Thus, the interactions were tested for statistical significance after controlling for all the other statistically significant predictors of basic reading (Caemmerer et al. 2018, 2023).

#### 2.3.5. Analysis Step 4: Cross-Battery Basic Reading and Writing Interrelations

Five models were tested to examine the interrelations between youths’ basic reading and writing performance. Each model also controlled for the statistically significant broad cognitive ability influences on academic skills. The cognitive–basic reading paths were established in step 2 of the current analysis and the cognitive–writing paths were established in a prior study (the paths from Gc, Gl, Gwm, Gs to broad writing were significant, Caemmerer et al. 2023). One model tested the influence of youths’ basic reading on their written expression skills, and another tested the influence of spelling on written expression skills. Written expression was estimated by 1 KTEA-II Written Expression, 1 WIAT-II Written Expression, 1 WIAT-III Essay Composition, and 1 WIAT-III Sentence Composition subtest. Spelling was estimated by two subtests (KTEA-II Spelling and WIAT Spelling). To avoid empirical under-identification, the variance of the WIAT Spelling subtest was fixed to the subtest’s unreliability (1—the reliability of the measure, as reported in the WIAT-III technical manual [NCS Pearson, 2010], multiplied by the subtest’s variance; Keith 2019).

Three alternative basic reading–spelling models were tested to determine the directionality of the effects (Keith 2019). One model tested a path from youths’ basic reading to their spelling, another tested a path from their spelling to their basic reading, and another tested bidirectional paths from their basic reading to spelling and from their spelling to basic reading (a non-recursive model).

#### 2.3.6. Model Evaluation

The individual models were evaluated according to several fit indices. The cut-off values that suggested good fit were the root mean square of error of approximation (RMSEA) values below 0.05, the standardized root mean residual (SRMR) values below 0.08, and the comparative fit index (CFI) and Tucker–Lewis index (TLI) values above 0.95 (Hu and Bentler 1999). The nested competing models were evaluated with the likelihood ratio test (Δ*χ*^2^). Statistically significant Δ*χ*^2^ supported the less constrained model with fewer degrees of freedom. The alternative non-equivalent models were compared with the adjusted Bayesian information criterion (aBIC), Akaike information criterion (AIC), and Bayesian information criterion (BIC). Smaller aBIC, AIC, and BIC values indicated better fit (Keith 2019). The effect sizes were evaluated with standardized regression coefficients; values greater than 0.05 were considered small, those greater than 0.10 were considered moderate, and those greater than 0.25 were considered large (Keith 2019).

## 3. Results

The data appeared normally distributed (skewness < 1.64 and kurtosis < 3.70) and the subtest means and standard deviations were similar to the normative samples (see Table 2).

### 3.1. Cognitive–Basic Reading Models

The fit of the cognitive–basic reading models ranged from acceptable to good (see models 1 and 2 in Table 3). The factor loadings of the four reading subtests on the basic reading latent variable factor were statistically significant and large, which suggests the subtests are generally good measures of basic reading. In the first model, paths from all the broad abilities were examined. Three paths were not statistically significant: visual processing (Gv β = 0.01, *SE* = 0.06, *p* = .91, 95% CI β = −0.11–0.12), processing speed (Gs β = 0.07, *SE* = 0.04, *p* = .12, 95% CI β = −0.02–0.15), and fluid reasoning (Gf β = −0.12, *SE* = 0.11, *p* = .27, 95% CI β = −0.34–0.08). These three paths were subsequently deleted.

**Figure 1:**
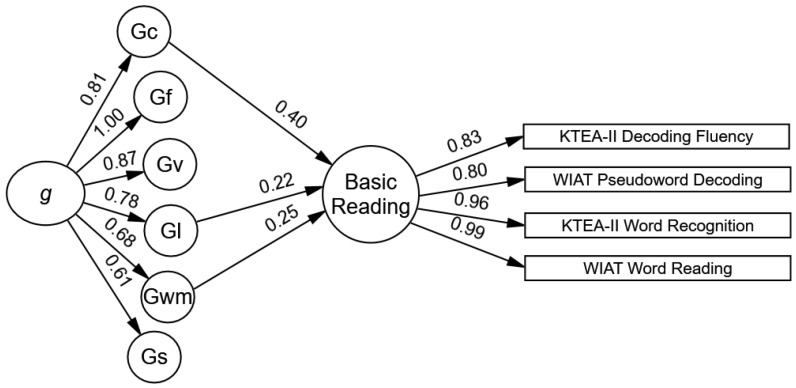
Model 2. The significant standardized estimates of cognitive influences on the youths’ basic reading. Note. The 66 cognitive subtests are not shown in this figure. See Table 1 for a list of the cognitive subtests and (Caemmerer et al. 2020) for a figure of the cross-battery CHC cognitive model.

The reduced model included statistically significant paths from verbal comprehension–knowledge, working memory, and learning efficiency, which were moderate to large in size (see Figure 1). These three cognitive–achievement paths were retained in all the other models. The largest standardized effect was from verbal comprehension–knowledge to basic reading (Gc β = 0.40, *SE* = 0.02, *p* < .001, 95% CI β = 0.36–0.45), which means that each standard deviation increase in the Gc resulted in a .40 standard deviation increase in the basic reading. The total indirect path from *g* to basic reading was statistically significant and large (*g* total indirect β = 0.66, SE = 0.01, *p* < .001, 95% CI β = 0.64–0.68).

The fit of the third model, which tested the direct effect from *g* to basic reading and did not include paths from the CHC broad cognitive abilities, was also acceptable to good (see Table 3 model 3). The fit of model 3 was worse than model 2 according to the likelihood ratio test, aBIC, AIC, and BIC. The standardized regression coefficient from *g* to basic reading was large (*g* β = 0.75, *SE* = 0.01, *p* < .001, 95% CI β = 0.73–0.77).

### 3.2. Developmental Differences in Cognitive–Basic Reading Relations

Five moderation models were tested to determine if the influence of Gc, Gv, Gwm, Gl, or Gs on basic reading varied across ages. One statistically significant interaction was found after controlling for the direct effects of age, the broad ability in the cross product, and the Gc, Gwm, and Gl paths that were significant in the final cognitive–basic reading model (model 2 in Table 3). Age moderated the effect of verbal comprehension–knowledge on basic reading (Gc *b* = 0.37, *SE* = 0.11, *p* < .01, 95% CI *b* = 0.14–0.56). Thus, the effect of Gc depends on youths’ age.

**Figure 2:**
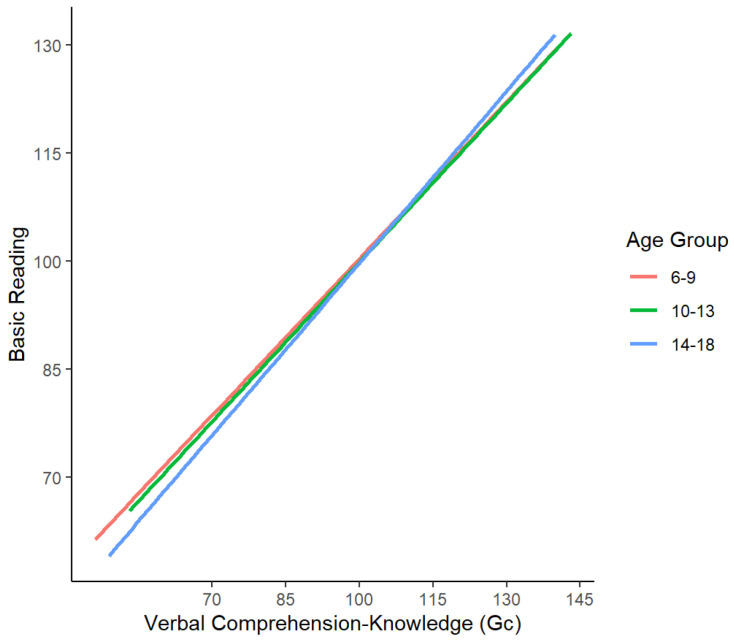
Statistically significant interaction between youths’ age and Gc–basic reading performance. Note. Interactions were tested with a continuous age variable, but youth were divided into age groups for illustrative purposes only.

For interpretation purposes, the interaction was graphed (Figure 2). For illustrative purposes only, the continuous age variable was divided into three age groups with relatively similar sample sizes: 6–9 (*n* = 1329), 10–13 (*n* = 1392), and 14–18 years old (*n* = 1206). Higher verbal comprehension–knowledge was associated with higher basic reading skills for all ages, particularly for those aged 14–18. Although the interaction was statistically significant, the graph illustrates that the difference in effects across ages is small.

### 3.3. Relations Between Children’s Basic Reading and Writing Skills

The fit of the basic reading–writing interrelations models were generally good (see Table 3), except the SRMR exceeded the cut-off value. Due to the poor SRMR, the residual correlations and modification indices were examined to identify the areas of misfit and the potential modifications to improve model fit (Keith 2019). Several residual correlations (the difference between the actual and implied correlation matrices) exceeded the cut-off of 0.10 in absolute value (Keith 2019). The largest residual correlations were between the WIAT and KTEA-II subtests. This misfit was likely due to the missing data patterns. None of the participants completed both the WIAT and KTEA-II; thus, maximum likelihood estimation was completely relied on to estimate these correlations based on the observed data from other variables. The changes suggested by the modification indices were not supported by theory or previous research. Thus, no model modifications were made. Despite the poor SRMR (0.10), subsequent models were interpreted due to the good fit of the CFI, TLI, and RMSEA.

#### 3.3.1. Basic Reading, Spelling, and Written Expression

**Figure 3:**
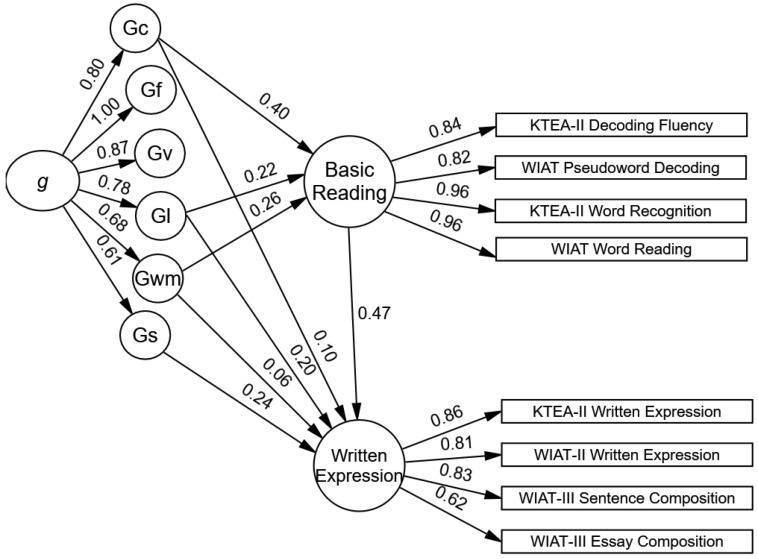
Model 4. Significant standardized influence of basic reading on written expression.

**Figure 4:**
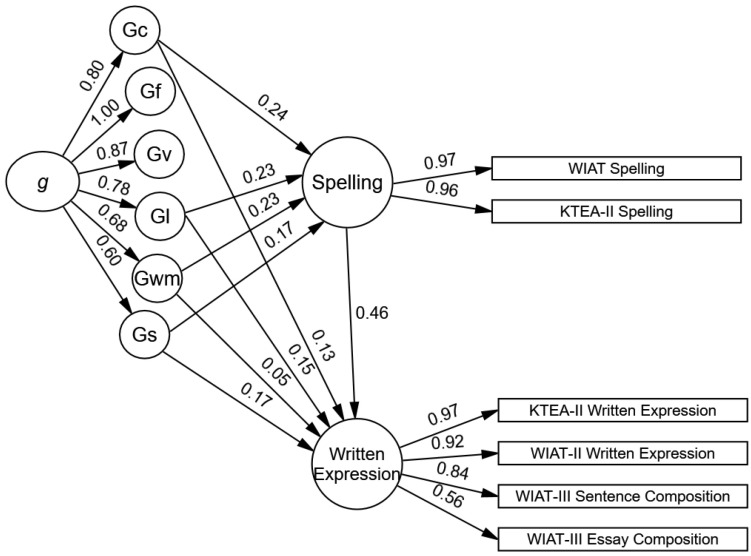
Model 5. Significant standardized influence of spelling on written expression. Note. The 66 cognitive subtests are not shown in the figures. See Table 1 for a list of the cognitive subtests and (Caemmerer et al. 2020) for a figure of the cross-battery CHC cognitive model.

The model fit was acceptable to good, with the exception of the SRMR (see models 4 and 5 in Table 3). The factor loadings of the written expression and spelling subtests on their latent factors were statistically significant and large (see Figure 3 and Figure 4). In model 4, the path from the youths’ basic reading to written expression was statistically significant and large (*b* = 0.43, *SE* = 0.03, *p* < .001, 95% CI *b* = 0.37–0.49). As illustrated in Figure 3, the influences of verbal–comprehension knowledge (total indirect β = 0.19, *SE* = 0.02, *p* < .001, 95% CI β = 0.15–0.23) and working memory (total indirect β = 0.12, *SE* = 0.02, *p* < .001, 95% CI β = 0.09–0.16) on written expression are primarily indirect through their direct effects on youths’ basic reading skills. The influence of learning efficiency on written expression was primarily direct (total indirect β = 0.10, *SE* = 0.02, *p* < .001, 95% CI β = 0.07–0.14). The total effects suggest the influences of verbal–comprehension knowledge (Gc) and learning efficiency (Gl) were similarly large sized, and the influence of working memory (Gwm) was moderate-sized and relatively smaller (Gl total effects β = 0.30, Gc total effects β = 0.29, Gwm total effect β = 0.18). The total indirect effect of *g* on written expression was large-sized (*g* total indirect effect β = 0.73, *SE* = 0.05, *p* < .001, 95% CI β = 0.64–0.82). The direct influence of the youths’ processing speed (Gs) on written expression was moderate-sized and the standardized coefficient was statistically significant (direct β = 0.24, *SE* = 0.05, *p* < .001, 95% CI β = 0.15–0.32), but the unstandardized coefficient was not statistically significant; yet, the confidence interval did not contain zero (*b* = 0.41, *SE* = 0.77, *p* = .59, 95% CI *b* = 0.24–0.85). The conflicting results suggest that the direct influence of Gs on written expression should be interpreted cautiously.

In model 5, the path from the youths’ spelling to their written expression was statistically significant and large (*b* = 0.46, *SE* = 0.04, *p* < .001, 95% CI *b* = 0.37–0.53). As illustrated in Figure 4, the influences of verbal–comprehension knowledge (total indirect β = 0.11, *SE* = 0.02, *p* < .001, 95% CI β = 0.08–0.15) and working memory (total indirect β = 0.11, *SE* = 0.02, *p* < .001, 95% CI β = 0.08–0.15) on written expression were primarily indirect through their direct effects on youths’ spelling skills. The influence of learning efficiency on written expression was primarily direct (total indirect β = 0.10, *SE* = 0.02, *p* < .001, 95% CI β = 0.08–0.15). The total effects suggest the influences of verbal–comprehension knowledge (Gc) and learning efficiency (Gl) are similar and large sized, and the influence of working memory (Gwm) is moderate-sized and relatively smaller (Gc and Gl total effects β = 0.25, Gwm total effect β = 0.16). The total indirect effect of *g* on written expression was large-sized (*g* total indirect effect β = 0.65, *SE* = 0.04, *p* < .001, 95% CI β = 0.62–0.76). The direct influence of youths’ processing speed (Gs) on their spelling and written expression was moderate-sized and the standardized coefficient was statistically significant (Spelling and Written Expression direct β = 0.17, *SE* = 0.04, *p* < .001, 95% CI Spelling β = 0.09–0.24, 95% CI Written Expression = 0.10–0.27), but the unstandardized coefficient was not statistically significant; yet, the confidence interval did not contain zero (Spelling *b* = 0.33, *SE* = 0.46, *p* = 0.48, 95% CI *b* = 0.16–0.68; Written Expression *b* = 0.34, *SE* = 0.73, *p* = 0.64, Written Expression 95% CI *b* = 0.18–0.77). The conflicting results suggest the direct influence of Gs on spelling and written expression should be interpreted cautiously.

#### 3.3.2. Basic Reading–Spelling

The fit for the three basic reading and spelling models was acceptable to good, with the exception of the SRMR (see models 6, 7, and 8 in Table 3). Three alternative models were compared to determine the directionality of the basic reading–spelling effect. In model 6, the path from basic reading to spelling was statistically significant and large (*b* = 0.85, *SE* = 0.04, *p* < .001, 95% CI *b* = 0.79–0.93); this basic reading–spelling path was the largest academic path of the three spelling models (see Figure 4). In model 7, the path from spelling to basic reading was statistically significant and large (*b* = 0.64, *SE* = 0.04, *p* < .001, 95% CI *b* = 0.57–0.72). Models 6 and 7 had the same degrees of freedom, so the likelihood ratio test could not be calculated, but model comparisons according to the aBIC, AIC, and BIC supported model 6 (see Table 3). Finally, in the bidirectional non-recursive model 8, the path from basic reading to spelling was statistically significant and large (*b* = 0.62, *SE* = 0.28, *p* < .05, 95% CI *b* = 0.01–1.07), but the path from spelling to basic reading was not statistically significant (*b* = 0.29, *SE* = 0.28, *p* = 0.31, 95% CI *b* = −0.47–0.63). The likelihood ratio test, aBIC, AIC, and BIC suggested that model 6 fit significantly better than model 8 (see Table 3), although it is worth noting the differences between the models were small. Thus, the results from the model comparisons are most supportive of the directionality of the effect from youths’ basic reading to their spelling skills and not vice versa.

**Figure 5:**
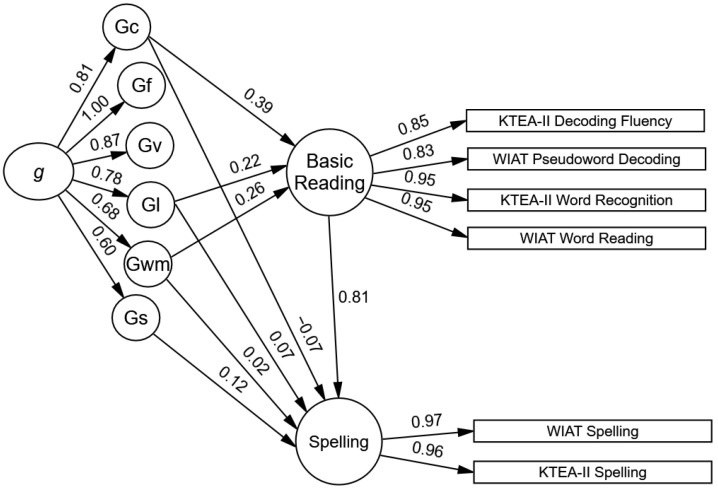
Model 5. The significant standardized influences of basic reading on spelling. Note. The 66 cognitive subtests are not shown in this figure.

Given model 6 is the final accepted model, further interpretation is warranted. As illustrated in Figure 5, the influences of verbal–comprehension knowledge (total indirect β = 0.32, *SE* = 0.02, *p* < .001, 95% CI β = 0.28–0.36), learning efficiency (total indirect β = 0.18, *SE* = 0.02, *p* < .001, 95% CI β = 0.14–0.23), and working memory (total indirect β = 0.21, *SE* = 0.02, *p* < .001, 95% CI β = 0.18–0.25) on spelling were primarily indirect through their direct effects on youths’ basic reading skills. The total effects suggest that the influences of these three cognitive abilities on spelling were similarly sized moderate-to-large effects (Gc and Gl total effects β = 0.25, Gwm total effect β = 0.24). The total indirect effect of *g* on spelling was large-sized (*g* total indirect effect β = 0.62, *SE* = 0.01, *p* < .001, 95% CI β = 0.60–0.65). The direct influence of youths’ processing speed (Gs) on spelling was moderate-sized and the standardized coefficient was statistically significant (direct β = 0.12, *SE* = 0.03, *p* < .001, 95% CI β = 0.06–0.18), but the unstandardized coefficient was not statistically significant; yet, the confidence interval did not contain zero (*b* = 0.23, *SE* = 0.33, *p* = 0.48, 95% CI *b* = 0.11–0.49). These conflicting results suggest that the direct influence of Gs on spelling should be interpreted cautiously.

## 4. Discussion

The purpose of this study was to examine the effects of children’s cognitive abilities on their basic reading skills, as well as the relations between their basic reading and writing achievement. This study used a cross-battery approach, utilizing many cognitive and academic subtests drawn from six intelligence tests and three academic achievement tests. The simultaneous analyses of data from many tests created comprehensively defined constructs, which allowed for the greater generalizability of the findings as compared to single-battery studies that may find spurious relations due to test idiosyncrasies.

### 4.1. Cognitive–Basic Reading Relations and Age Moderation

Consistent with previous research, youths’ general intelligence, verbal comprehension–knowledge (Gc), and working memory (Gwm) significantly influenced their basic reading skills in our study. General intelligence (*g*) had the largest total effect on their basic reading skills (Benson et al. 2016; Caemmerer et al. 2018; Zaboski et al. 2018). The influence of general intelligence on basic reading was mediated by the direct effects from three CHC broad abilities. Among the CHC broad cognitive abilities, the youths’ verbal–comprehension knowledge (Gc) had the largest direct effect. Youth with stronger acquired knowledge of vocabulary, language, and general information performed better on word recognition and decoding tasks. Like most of the prior studies that examined developmental differences, Gc’s effect appeared to slightly increase with age (Benson 2008; Hajovsky et al. 2014; McGrew and Knopik 1993; Zaboski et al. 2018). This suggests that Gc may become slightly more important in adolescence when youth must recognize and sound out more advanced words. The youths’ working memory (Gwm) also had a large direct effect on their basic reading skills. Youth with a better developed ability to mentally hold and manipulate information in their immediate awareness scored higher on basic reading tasks. In our study, we found that Gwm had a stable influence on the youths’ basic reading skills from ages 6 to 18, which aligns with prior CHC-based studies, but contradicts a correlational meta-analysis that found a stronger effect prior to fourth grade (Caemmerer et al. 2018; Floyd et al. 2008; Hajovsky et al. 2014; Peng et al. 2019). The significant Gc– and Gwm–basic reading relations were consistently found in prior studies with a variety of tests (Caemmerer et al. 2018; Elliott et al. 2010; Floyd et al. 2008; Hajovsky et al. 2014; McGrew and Knopik 1993), suggesting that the measurement differences had little influence on the importance of these cognitive abilities.

Due to previous inconsistent findings, we tested the direct influence of all the other CHC cognitive broad abilities on basic reading. Our study found that youths’ ability to learn, store, and retrieve new information in long-term memory had a direct moderate-sized influence on their basic reading skills. The influence of learning efficiency (Gl) has been examined less often in previous research because some studies did not test this direct path and some intelligence tests do not measure Gl. Single test studies with the WJ tests produced mixed Gl-basic reading findings (Floyd et al. 2008; McGrew and Knopik 1993; Niileksela et al. 2016), and the single study with the KABC-II and KTEA-II found a significant Gl-basic reading relation (Hajovsky et al. 2014). The importance of Gl was demonstrated in our cross-battery study that included Gl subtests from the KABC-II, DAS-II, and WJ III. A few studies suggested that the Gl-basic reading relation may be stronger for younger children (Floyd et al. 2008; Hajovsky et al. 2014), but our study found the relation was stable across ages 6 to 18.

Our findings suggest that the three other broad cognitive abilities did not directly influence youths’ basic reading achievement. Although previous WJ studies found that youths’ processing speed (Gs) was a significant predictor (Floyd et al. 2008; McGrew and Knopik 1993; Niileksela et al. 2016), our cross-battery study and single-test studies with the WISC-V and DAS-II did not find a Gs-basic reading relation (Caemmerer et al. 2018; Elliott et al. 2010). Thus, it seems likely that the influence of Gs may vary based on measurement differences between the WJ and other tests. Lastly, similar to most prior studies, fluid reasoning (Gf) and visual processing (Gv) did not have significant direct influences on basic reading in our study (Caemmerer et al. 2018; Floyd et al. 2008; Hajovsky et al. 2014; Niileksela et al. 2016; Zaboski et al. 2018). It is important to note that the studies that examined the influence of cognitive abilities separately, rather than analyzing the influence of several cognitive abilities together, may have found a significant effect for some of the non-significant predictors in our study. For example, a meta-analysis of the correlations between Gf and basic (or code) reading skills found a small significant effect (*r* = 0.08, Peng et al. 2019). When several broad cognitive abilities are examined together, such as in our study, the analyses accounted for the covariances between the six CHC broad cognitive abilities and allowed for indirect effects. The direct influence of Gf, for example, was not statistically significant in our model after Gf’s relations with the other significant broad cognitive abilities were taken into account. It is also worth noting again that Gf and *g* were statistically indistinguishable in this study, as in other studies (Gustafsson 1984; Reynolds et al. 2013). Thus, the finding of the large total indirect effect of *g* on basic reading could also be interpreted as a large effect of Gf on basic reading.

The three significant predictors in this basic reading study (Gc, Gwm, Gl) also had significant influences on youths’ broad writing skills (a factor that combined spelling and written expression) in another cross-battery study with the same sample (Caemmerer et al. 2023). These convergent findings suggest these three cognitive abilities are important for understanding language-based academic skills (Shanahan 2006). The strength of working memory and learning efficiency’s influence on youths’ basic reading and broad writing skills were similarly sized, but the relation between verbal–comprehension knowledge (Gc) and basic reading was substantially stronger than the Gc-broad writing relation. Additionally, processing speed had a moderate influence on the youths’ broad writing but no direct effect on basic reading, suggesting there are some unique differences between the two language-based academic skills.

### 4.2. Basic Reading–Writing Interrelations

#### 4.2.1. Basic Reading, Spelling, and Written Expression

Similar to previous literature, our study supported the effect of basic reading and spelling skills on written expression (Andersen et al. 2018; Kim et al. 2015; Mäki et al. 2001; Parkin et al. 2020). Even after controlling for the influence of cognitive abilities, we found that the youths’ basic reading and spelling skills had a strong influence on their advanced writing skills. As children learn to decode and recognize words and to spell, they become more competent at writing sentences and essays. Interestingly the strength of the basic reading–written expression and spelling–written expression relations was nearly identical for these two different mediators.

#### 4.2.2. Basic Reading–Spelling

Previous research has been divided with respect to the direction of the relation between basic reading and spelling. In our study, we tested three alternatives—a direct effect from basic reading to spelling, a direct effect from spelling to basic reading, and bidirectional direct effects. The fit of the direct effect from basic reading to spelling model was superior to the other two models, and only the path from basic reading to spelling was significant in the bidirectional model and not vice versa. Altogether, the findings more strongly supported the unidirectional effect of youths’ basic reading on their spelling. That is, as youths become more skilled in reading words, they also become more proficient at encoding words accurately. This finding is consistent with other studies conducted with school-aged children (Ahmed et al. 2014; Kim and Park 2018; Schaars et al. 2017). While some prior studies have found a unidirectional effect of spelling on reading, these studies are almost exclusively focused on preschool to early elementary students and most did not examine both directions of the effects (Andersen et al. 2018; Caravolas et al. 2001; Clemens et al. 2014; Hand et al. 2022; McNeill et al. 2023; Martins et al. 2013). This discrepancy in findings could be indicative of age as a moderator of the basic reading–spelling relation. Further research, with samples inclusive of younger children, is needed to clarify the role of age in the relations between basic reading and spelling.

### 4.3. Limitations and Future Directions

This study had some limitations that are important to consider alongside our findings. The data used for this analysis are cross-sectional, meaning that our models cannot take temporal precedence into account. Longitudinal data are preferable for tests of mediation (Maxwell et al. 2011; Preacher 2015), such as our tests of the directionality of the basic reading–spelling relation and the indirect influences of youths’ basic reading and spelling on their written expression performance. Future studies that incorporate longitudinal data may provide stronger evidence of directionality and the magnitude of the mediation effects. Additionally, we tested age moderation in our study, but future research should continue to examine the generalizability of cognitive–achievement relations across other characteristics, such as gender, race and ethnicity, cognitive ability levels, and academic skill levels (Caemmerer et al. 2024; Hajovsky et al. 2014, 2018, 2023; Woods et al. 2021).

Another limitation was the amount of missing data, which is inherent in cross-battery analyses of several tests. As illustrated in Table 2, some intelligence and academic subtests had a high degree of missingness and there was no overlapping WIAT and KTEA-II data because no participant completed both academic tests. We used full information maximum likelihood estimation (FIML) to handle the missing data in our study. While research suggests that FIML may be robust to high amounts of missing data if large sample sizes are used in planned missingness designs (Graham et al. 2006; Zhang and Yu 2021), further research is needed to better understand its possible influence on the results and model fit, particularly the SRMR.

Furthermore, although many subtests were included in our cross-battery study, analyses were limited to the constructs measured by those tests. It was not possible to include phonemic awareness (which is similar to the CHC broad cognitive ability auditory processing, Ga) in our analyses because the WJ III was the only test that included Ga subtests. Auditory processing has an important influence on reading and spelling skills (Niileksela et al. 2016; Strattman and Hodson 2005) and its inclusion may have affected the magnitude of the effects in our study; thus, future research should incorporate multiple tests that measure phonemic awareness.

This limitation also extends to the academic constructs we examined in our study. Basic reading tasks involve reading pseudo-words or non-words and reading real words. There is some evidence to suggest real-word reading and non-word decoding skills follow differing developmental trajectories, particularly in poor readers (Steacy et al. 2021). Also, cognitive predictors, such as language abilities (specifically vocabulary), may differentially influence real-word and pseudo-word reading performance (Ricketts et al. 2007). Due to the limited number of basic reading subtests included in our sample, we were unable to explore these narrower potential relations. Future research should further subdivide basic reading skills into pseudo-word and real-word reading factors.

Finally, we recognize that some theorists do not differentiate between cognitive ability and academic achievement tests (Humphreys 1973). Many researchers, test developers, and practitioners, however, find the distinction useful and evidence suggests that cognitive ability and academic achievement are highly related but distinct constructs (Deary et al. 2007; Kaufman et al. 2012). Furthermore, evidence has shown that cognitive abilities are the leading indicators of academic skill attainment and not vice versa (Reynolds and Turek 2012; Watkins et al. 2007).

## 5. Conclusions

Youths’ basic reading skills appear to be influenced by their general intelligence, verbal comprehension knowledge, and, to a lesser extent, working memory and learning efficiency. A unique contribution of our study is the revelation that most of the broad cognitive ability–basic reading relations were stable between the ages of 6 to 18. The only exception was verbal–comprehension knowledge, for which there was a significant but small developmental effect. It seems that verbal comprehension knowledge exerts a greater effect on basic reading skills in older children and adolescents, but the size of this interaction is small. Youths’ basic reading skills strongly influence both their written expression and spelling performance, and the effect is larger on spelling. A unidirectional effect of basic reading on spelling fits the data best as compared to a unidirectional effect of spelling on basic reading or a bidirectional effect between the two academic skills. Our cross-battery study examined cognitive–basic reading and reading–writing relations with broader coverage and depth, thus circumventing measurement differences across tests. Our findings also contributed important validity evidence for youths’ intelligence scores (American Educational Research Association et al. 2014) by expanding the evidence base for the relations between youths’ cognitive abilities on their basic reading and writing performance.

## Figures and Tables

**Table 1:** Sample sizes for the seven samples. nine tests, and the overlap in tests between samples.

Tests	KABC-II	WJ III	WISC-III	WISC-IV	WISC-V	DAS-II	KTEA-II	WIAT-II	WIAT-III
KABC-II XBA	347	89	123	58	-	-	-	-	-
KABC-II/KTEA-II	2223	-	-	-	-	-	2223	-	-
WISC-IV/DAS-II	-	-	-	202	-	202	-	-	-
DAS-II/WIAT-II	-	-	-	-	-	370	-	370	-
WISC-IV/WIAT-II	-	-	-	532	-	-	-	532	-
WISC-V/WIAT-III	-	-	-	-	181	-	-	-	181
WISC-V/KABC-II	88	-	-	-	88	-	-	-	-
Total	2658	89	123	792	269	572	2223	902	181

Note. This table is adapted from (Caemmerer et al. 2020) and a similar version appears in (Caemmerer et al. 2023).

**Table 2:** Details for the cognitive and academic subtests from the 9 tests.

Achievement Tests	*N*	*Mean*	*SD*
**WIAT (Edition; Specific Achievement Skill)**			
Essay Composition (III; Written Expression)	151	100.76	15.72
Pseudo-word Decoding (II and III; Basic Reading)	1054	102.20	13.67
Spelling (II and III; Basic Writing)	1083	101.59	14.26
Sentence Composition (III; Written Expression)	179	99.59	12.83
Word Reading (II and III; Basic Reading)	1078	101.92	14.28
Written Expression (II; Written Expression)	871	101.91	15.49
**KTEA-II (Specific Achievement Skill)**			
Decoding Fluency (Basic Reading)	2021	99.88	15.05
Spelling (Basic Writing)	2021	99.74	14.91
Word Recognition (Basic Reading)	2223	99.83	14.66
Written Expression (Written Expression)	2223	100.03	15.16
**Cognitive Tests**			
**DAS-II (Broad Ability; Narrow Ability)**			
Copying (Gv; Visualization)	178	51.23	8.41
Digits Backward (Gwm; Working Memory)	557	49.82	8.56
Digits Forward (Gwm; Memory Span)	557	49.81	9.78
Early Number Concepts (Gf; Quantitative Reasoning)	178	51.10	8.64
Matching Letter Like Forms (Gv; Visualization)	178	51.71	9.08
Matrices (Gf; Induction)	557	50.21	9.19
Naming Vocabulary (Gc; Lexical Knowledge)	178	51.35	9.41
Pattern Construction (Gv; Visualization)	557	49.97	8.65
Rapid Naming (Gs; Rate of Test-Taking)	557	50.47	8.97
Recall of Designs (Gv; Visual Memory)	556	50.25	8.78
Recognition of Pictures (Gv; Visual Memory)	557	50.26	9.29
Recall of Objects-Immediate (Gl; Free Recall Memory)	557	49.06	10.32
Recall of Objects-Delayed (Gl; Free Recall Memory)	557	50.17	9.46
Recall of Sequential Order (Gwm; Working Memory)	557	50.09	9.48
Sequential and Quantitative Reasoning (Gf; Quantitative Reasoning)	556	50.55	9.09
Speed of Information Processing (Gs; Perceptual Speed)	557	51.07	9.21
Verbal Comprehension (Gc; Listening Ability; cross-loading: Gf)	178	50.15	8.96
Verbal Similarities (Gc; Lexical Knowledge)	557	50.66	8.48
Word Definitions (Gc; Lexical Knowledge)	556	50.19	8.84
**KABC-II (Broad Ability; Narrow Ability)**			
Atlantis (Gl; Associative Memory)	2654	10.02	3.09
Atlantis Delayed (Gl; Associative Memory)	2435	9.93	2.80
Block Counting (Gv; Visualization)	2655	9.97	3.00
Expressive Vocabulary (Gc; Lexical Knowledge)	2656	9.85	2.95
Gestalt Closure (Gv; Closure Speed; cross-loading: Gc)	619	10.00	2.89
Hand Movements (Gwm; Memory Span; cross-loading: Gf)	2656	10.08	2.88
Number Recall (Gwm; Memory Span)	2657	10.24	2.86
Pattern Reasoning (Gf; Induction)	2656	10.19	2.96
Rebus (Gl; Associative Memo)	2657	10.14	3.04
Rebus Delayed (Gl; Associative Memo)	2407	10.03	2.96
Riddles (Gc; Lexical Knowledge)	2657	10.14	3.04
Rover (Gv; Spatial Scanning)	2652	10.15	3.02
Story Completion (Gf; General Sequential Reasoning)	2653	10.10	2.98
Triangles (Gv; Visualization)	2656	10.00	2.91
Verbal Knowledge (Gc; Lexical Knowledge)	2657	10.01	2.94
Word Order (Gwm; Memory Span)	2657	9.93	2.83
**WISC (Broad Ability; Narrow Ability; Edition)**			
Arithmetic (Gwm; Working Memory; cross-loaded: Gf, Gs; III–V)	880	10.32	2.77
Block Design (Gv; Visualization; III–V)	1178	10.18	2.84
Cancelation (Gs; Perceptual Speed; IV–V)	998	10.03	3.02
Coding (Gs; Rate of Test Taking; III–V)	1178	10.06	2.90
Comprehension (Gc; General Verbal Information; III–V)	1174	10.25	2.89
Digit Span (Gwm; Memory Span and Working Memory; III–V)	1167	10.04	2.86
Figure Weights (Gf; Quantitative Reasoning; V)	269	9.92	2.69
Information (Gc; General Verbal Information; III–V)	1124	10.25	2.84
Letter-Number Sequencing (Gwm; Working Memory; III–V)	1050	10.00	2.82
Matrix Reasoning (Gf; Induction; IV–V)	1060	10.21	2.86
Object Assembly (Gv; Closure Speed; III)	123	10.34	2.95
* Picture Arrangement (Gf; General Sequential Reasoning; III)	123	10.65	3.45
Picture Completion (Gv; Flexibility of Closure; cross-loaded: Gc; III and IV)	324	10.33	3.00
Picture Concepts (Gf; Induction; IV–V)	1060	10.28	2.92
Picture Span (Gwm; Working Memory; V)	269	9.75	2.66
Similarities (Gc; Lexical Knowledge; III–V)	1179	10.18	2.87
Symbol Search (Gs; Perceptual Speed; III–V)	1143	10.21	2.93
Visual Puzzles (Gv; Visualization; V)	268	10.06	2.62
Vocabulary (Gc; Lexical Knowledge; III–V)	1178	10.14	2.91
**WJ III (Broad Ability; Narrow Ability)**			
Analysis-Synthesis (Gf; General Sequential Reasoning)	87	102.91	17.19
Auditory Working Memory (Gwm; Working Memory)	88	105.40	13.91
Concept Formation (Gf; Induction)	89	105.36	13.90
Decision Speed (Gs; Perceptual Speed)	88	100.56	16.18
General Information (Gc; General Verbal Information)	89	98.37	16.16
Numbers Reversed (Gwm; Working Memory)	89	100.62	14.31
Picture Recognition (Gv; Visual Memory; cross-loaded: Gl)	89	100.79	12.58
Spatial Relations (Gv; Visualization)	89	100.62	11.33
Verbal Comprehension (Gc; Lexical Knowledge)	89	102.55	14.24
Visual-Auditory Learning (Gl; Associative Memory)	89	94.65	19.76
Visual Matching (Gs; Perceptual Speed)	89	95.84	13.37

Note. This table is adapted from (Caemmerer et al. 2023). Six cognitive subtests were cross-loaded on more than one CHC broad ability based on prior studies (Reynolds et al. 2013; Caemmerer et al. 2020). * The narrow ability classification of this subtest is based on our opinion.

**Table 3:** Model fit for cognitive–achievement models.

Model	χ2 (df)	Δχ2 (Δdf)	Δp	CFI /TLI	RMSEA	SRMR	aBIC	AIC	BIC
1. All 6 Broad Cognitive Ability Paths to Basic Reading	2890.05 (1474)	-	-	0.96	0.02	0.09	370,849.32	370,133.66	371,583.33
2. Significant Broad Cognitive Ability Paths to Basic Reading	2894.10 (1477)	4.05 (3)	0.26	0.96	0.02	0.09	370,838.07	370,131.71	371,562.55
3. g Direct Effect to Basic Reading	3108.95 (1478)	214.85	<.001	0.95	0.02	0.09	371,047.83	370,344.56	371,769.13
4. Basic Reading to Written Expression	3092.73 (1582)	-	-	0.96	0.02	0.10	397,013.72	396,254.69	397,792.22
5. Spelling to Written Expression	2956.22 (1502)	-	-	0.96/0.95	0.02	0.10	373,701.52	372,961.08	374,460.96
6. Basic Reading to Spelling	3097.67 (1551)	-	-	0.96	0.02	0.10	393,053.64	392,316.30	393,809.90
7. Spelling to Basic Reading	3100.40 (1551)	-	-	0.96	0.02	0.10	393,056.37	392,319.03	393,812.63
8. Bidirectional Basic Reading–Spelling Paths (Nonrecursive)	3096.22 (1550)	1.46 (1)	0.23	0.96	0.02	0.10	393,057.28	392,316.84	393,816.72

## Data Availability

The data were obtained from Pearson Assessments; we cannot share the data with others. Requests for an Pearson Assessments standardization data license can be submitted at https://www.pearsonassessments.com/forms/standardization-data-license-requests.html (accessed on 15 June 2024). The standardization data were from the Wechsler Intelligence Scale for Children, Fourth Edition (WISC-IV), copyright © 2003 NCS Pearson, Inc, were used with permission. All the rights are reserved. The standardization data are from the Wechsler Intelligence Scale for Children, Fifth Edition (WISC-V), copyright © 2014 NCS Pearson, Inc. These were used with permission. All the rights are reserved. The standardization data are from the Differential Ability Scales, Second Edition (DAS-II), copyright © 1998, 2000, 2004, 2007 NCS Pearson, Inc. and Colin D. Elliott. The normative data, copyright © 2007 NCS Pearson, Inc., were used with permission. All the rights are reserved. Standardization data from the Wechsler Individual Achievement Test, Second Edition (WIAT-II), copyright © 2001 NCS Pearson, Inc., were used with permission. All the rights are reserved. The standardization data from the Wechsler Individual Achievement Test, Third Edition (WIAT-III), copyright © 2009 NCS Pearson, Inc., were used with permission. All the rights are reserved. The standardization data from the Kaufman Assessment Battery for Children, Second Edition (KABC-II), copyright © 2004 NCS Pearson, Inc., were used with permission. All the rights are reserved. The standardization data from the Kaufman Test of Individual Achievement, Second Edition Comprehensive Form (KTEA-II Comprehensive Form), copyright © 2004 NCS Pearson, Inc., were used with permission. All rights reserved.
